# Surface conversion of the dynamics of bacteria escaping chemorepellents

**DOI:** 10.1140/epje/s10189-024-00450-7

**Published:** 2024-09-15

**Authors:** Asma Braham, Laurence Lemelle, Romain Ducasse, Houyem Toukabri, Eleonore Mottin, Benoit Fabrèges, Vincent Calvez, Christophe Place

**Affiliations:** 1 Laboratoire de Géologie de Lyon-Terre Planètes Et Environnement, ENS de Lyon, University Claude Bernard, CNRS, 69342 Lyon, France; 2 Laboratoire de Physique, ENS de Lyon, CNRS, 69342 Lyon, France; 3 Laboratoire Jacques-Louis Lions, Université Paris Cité, Sorbonne University, CNRS, 75005 Paris, France; 4 Centre for Genomic Regulation, C/ Dr Aiguader, 88, 08003 Barcelone, Spain; 5 Institut Camille Jordan, University Claude Bernard, CNRS, 69100 Villeurbanne, France

## Abstract

**Abstract:**

Flagellar swimming hydrodynamics confers a recognized advantage for attachment on solid surfaces. Whether this motility further enables the following environmental cues was experimentally explored. Motile *E. coli* (OD ~ 0.1) in a 100 µm-thick channel were exposed to off-equilibrium gradients set by a chemorepellent Ni(NO_3_)_2_-source (250 mM). Single bacterial dynamics at the solid surface was analyzed by dark-field videomicroscopy at a fixed position. The number of bacteria indicated their congregation into a wave escaping from the repellent source. Besides the high velocity drift in the propagation direction within the wave, an unexpectedly high perpendicular component drift was also observed. Swimming hydrodynamics CW-bends the bacteria trajectories during their *primo* approach to the surface (< 2 µm), and a high enough tumbling frequency likely preserves a notable lateral drift. This comprehension substantiates a survival strategy tailored to toxic environments, which involves drifting along surfaces, promoting the inception of colonization at the most advantageous sites.

**Graphical abstract:**

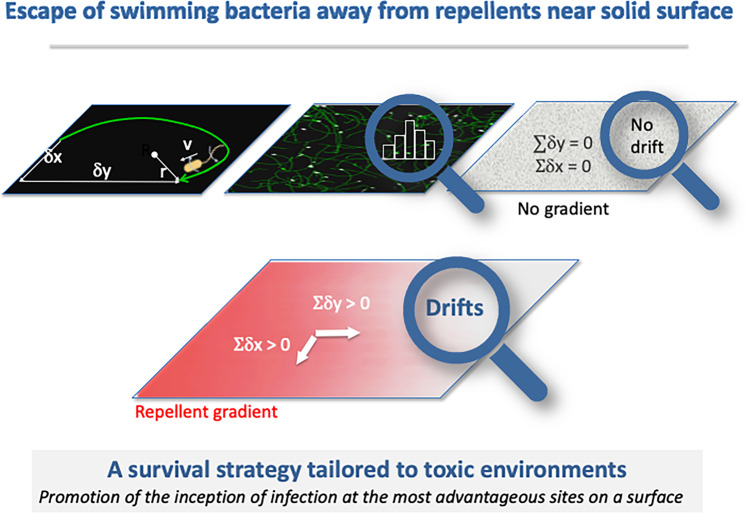

**Supplementary Information:**

The online version contains supplementary material available at 10.1140/epje/s10189-024-00450-7.

## Introduction

Microorganisms survivability and infectivity are enhanced by their ability to attach onto solid surfaces to form complex communities [1–6]. Within these communities, microorganisms are protected from inhospitable environmental variations and destruction by antibiotics and disinfectants. Flagellar swimming is characterized by a run-and-tumble motion [7, 8]. It confers to bacteria approaching a solid surface a recognized propitious advantage for attachment [9]. Hydrodynamic interactions between a propelled bacterium and a solid surface effectively confine a swimming population to the vicinity of the surface as evidenced by the high bacterial concentration [10] and long residence time [11] at the surface. Hydrodynamics coupled with thermal agitation amplifies the temporal fluctuations of the distance between a swimming bacterium and a solid surface [12]. It increases the rates of occasional contacts with the surface at molecular distance such as those of fimbriae, promoting reversible attachment [13] that are essential in mediating the transition from a free-living planktonic population to a sessile lifestyle [14]. Besides, hydrodynamics coupled with steric constraints hinders half of the tumbles that are less efficient than those in the bulk, and conversely, the remaining half destabilizes more significantly the swimming direction [15]. It facilitates surface reorientations and escapes towards the bulk [15–17] which argues in favor of the possibility of intricate sub-surface navigation.

Swimming is an effective way for *E. coli* to navigate towards preferential regions in the bulk [18]. In bulk, *E. coli* performs chemotaxis via a run-and-tumble strategy wherein sensitive temporal comparisons lead to a biased random walk characterized by longer runs in the preferred gradient direction [19]. Whether the changes in swimming behavior near surfaces maintain this advantage remains debated, as opposed to specific surface motilities like twitching, gliding, and sliding [2][20–22]. Bacteria redistributions at the agar gel surface, dependent on whether bacteria are motile or chemosensitive, along with their two-dimensional modeling as a chemotactic response to steep local spatial gradients produced by growth, inferred that flagellar swimming provides the advantage of surface navigation towards preferential regions [23, 24]. However, in these two-dimensional crowded environments, flagellar swimming significantly differs from the run-and-tumble behavior observed at a solid/liquid interface. At high cell densities, hydrodynamic interactions between swimmers near a solid/liquid interface hinder chemotactic behavior [25]. Besides, the run-and-tumble approach of a polymer/liquid interface is markedly different from that of a solid/liquid interface [26]. Solid surfaces are well known to drastically alter the bulk run-and-tumble walk of a bacterium. A self-propelled *E. coli* swimming at low Reynolds number tends to circle as it reaches interfaces [5]. The oriented clockwise circular motion is explained by hydrodynamics, due to an increased opposite drag on the body and the flagella. Its trajectory’s curvature increases while its swimming speed decreases as it approaches the surface [27–30]. This circling motion hampers bacteria ability to explore its surface environment by reducing its effective diffusion coefficient [31]. However, bacteria tightly confined between two surfaces exhibit a superdiffusive behavior which becomes more pronounced in a chemoattractant gradient [32], indicating that nontrivial distributions of swimming bacteria can emerge from simple physical gradients in the level of confinement [33]. At the population scale, the bacteria displacement toward preferential conditions has been described phenomenologically by a drift velocity, which complements the random motion of bacteria [34]. In particular, the drift velocity of individual *E. coli* bacteria has been measured to diminish to zero near surfaces in a gradual, shallow linear chemical gradient that aligns with the surface [35]. Therefore, it is expected that the presence of solid surfaces will cancel out the chemotactic drift component of *E. coli*, although this surface effect has not been thoroughly investigated with chemorepellents.

In this study, we generate an off-equilibrium chemorepulsive gradient within a microfluidics channel and study the average behavioral chemorepellent response at a fixed position on the surface over time. A wave was triggered using a nickel nitrate solution as the strong *E. coli* chemorepulsive response to Ni^2+^ cations and NO_3_^−^ anions is well-documented [36, 37]. The low bacteria concentration in the wave was appropriate to track single bacteria, and describe the individual dynamics. Here we show that, contrary to expectation, in the wave, the bacteria population exhibits along with a notable high velocity drift in the propagation direction, an unexpectedly high perpendicular component drift prompting discussion on surface colonization strategies.

## Materials and methods

### Bacterial strains

*E. coli* strain AW574 (*str*^*r*^ [38]), which is considered a wild type for motility and chemotaxis, was used [18].

### Medium and growth conditions

Colonies of *E. coli* strain AW574 were obtained by plating frozen *E. coli* glycerol stocks on agar plates that were incubated overnight at 37 °C. A volume of 2 mL of TN medium (4 g L^−1^ Tryptone, 2.5 g L^−1^ NaCl, 0.4% [vol] glycerol, 25 µg mL^−1^ streptomycin) was inoculated with a single colony, and saturated cultures were grown overnight using a rotary shaker (300 rpm) in 14 mL sterile, polypropylene tubes at 32 °C. The overnight culture was used to inoculate 2 ml of the TN medium at 0.05 OD at 600 nm and grown again at 32 °C and 300 rpm for 4 h. A 200 µl aliquot of the culture in the exponential phase was then diluted to 1:10 in 2 mL at 32 °C with a fresh TN medium. The solution was left for 1-h culture at 32 °C and 300 rpm to reach an OD at 600 nm ~ 0.09 to 0.11. Bacteria were pipetted once (without reflux) from the surface with a cut tip to reduce the shearing of the cell and ensure maximal motility.

### Microfluidic channel

**Fig. 1:**
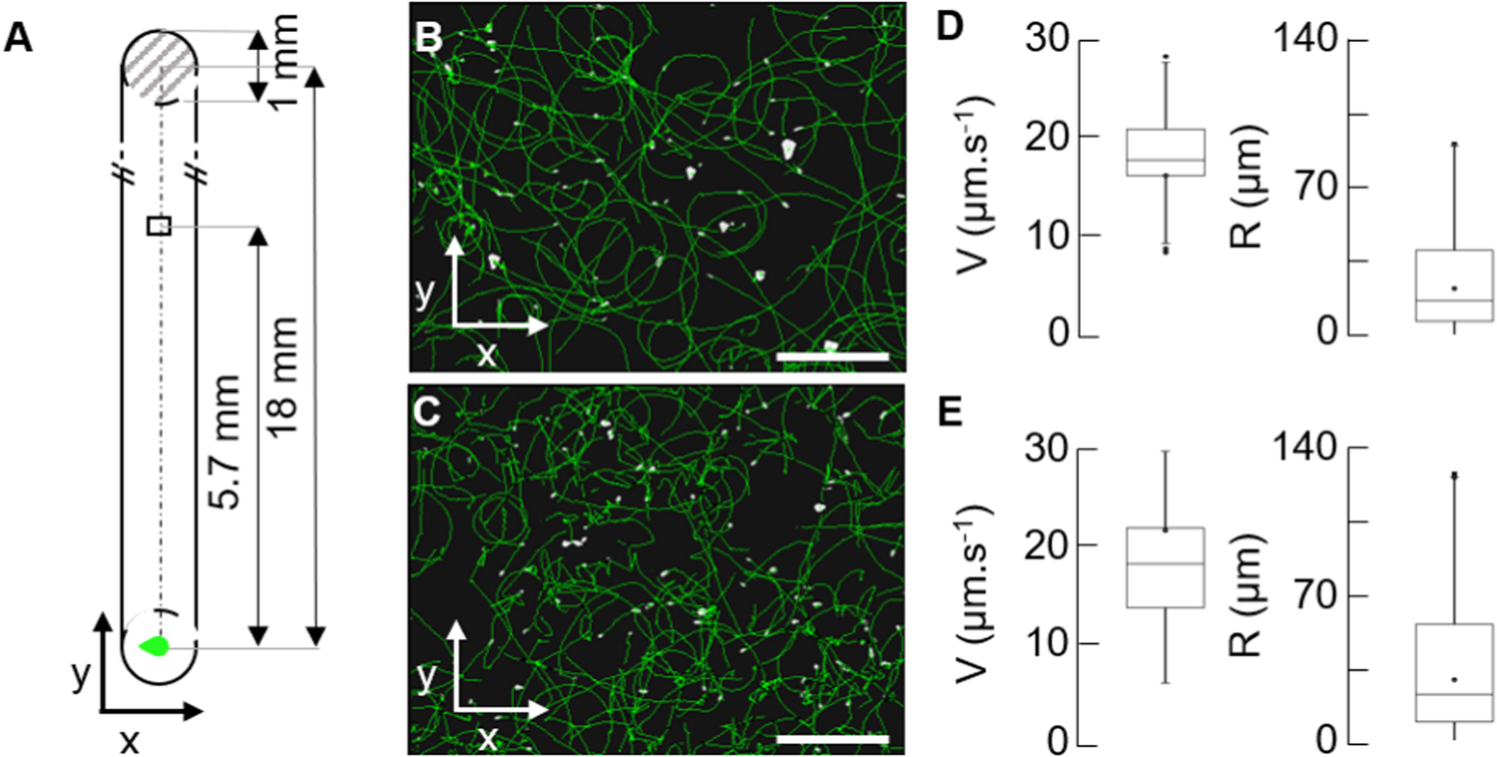
Tracking of bacteria swimming near a solid surface **A** Schematic representation (to scale) of a microfluidic channel. One well is sealed (dashed grey) and the other is filled with a repellent solution (green). The field of view (294 µm × 221 µm) at the fixed position y = 5.7 mm of observation is drawn. **B–C** The circular trajectories (green) of the swimming *E. coli* near the surface followed by 2D tracking microscopy during 10 s, are superimposed on the bacteria (white) of the first frame of the acquisition at 450 s, before the passage of the wave (**B**), and at 1290 s, in the wave (**C**). Scale bar = 50 µm. **D** Box plots showing median V and median R of the 65 trajectories of *E. coli* swimming near the surface of (**B**). Center lines show medians, and edges show the first (Q25) and third quartiles (Q75). “Whiskers” extend to the largest and smallest data points within 1.5 interquartile ranges of the first and third quartiles. **E** same than (**D**) for the 231 trajectories of (**C**)

One channel (17 mm × 1 mm × 0.1 mm, L × W × h) of a μ–Slide VI 0.1 flow chamber (IBIDI) was filled with 5 µL bacteria solution and then sealed at one extremity. The well at the opposite extremity was filled with 4 µl of 250 mM Ni(NO_3_)_2_ (Acros OrganicsTM, C.A.S.: 13478-00-7, MW: 290.8 g mol^−1^, purity 99%) (Fig. 1A). This μ–Slide was mounted on an inverted microscope stage at room temperature (23 °C) to observe the surface at a fixed position: 5.7 mm from the center of the well.

### Acquiring images

**Fig. 2:**
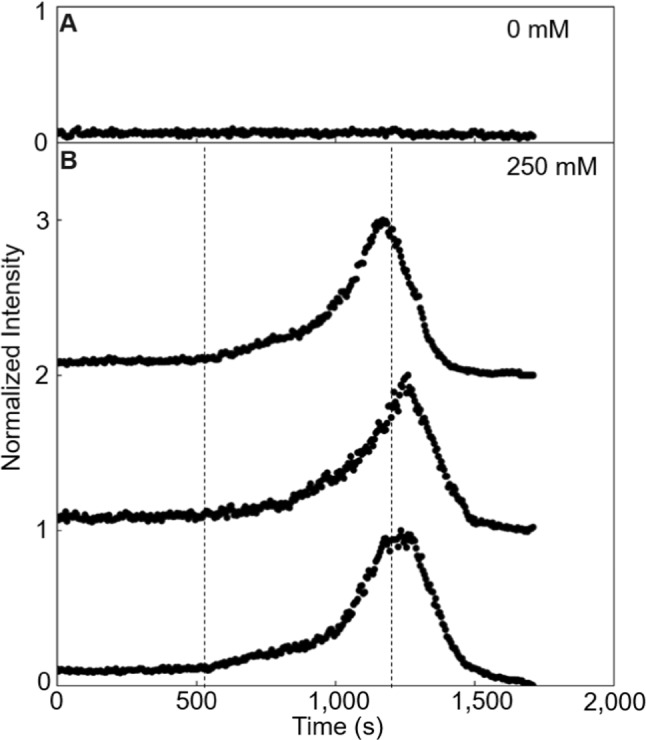
Control and triplicate of the passage of an *E. coli* wave at the surface **A** Integrated intensities of an image versus time of the bacteria at the surface of the microfluidic channel in the absence of repellent addition. **B** Triplicates of the integrated intensities of an image versus time of bacteria swimming at the surface of the microfluidic channel y = 5.7 mm after repellent addition at t = 0 s (circle). The integrated intensity is normalized versus the maximum intensity of each curve. Triplicated curves are shifted vertically by an interval of 1. Concentrations of the Ni(NO_3_)_2_ solution are indicated in the top right of each box. The first dashed vertical line represents the limit between phase #1 and phase #2. The second dashed line represents the average mean of the three peak times

The bacteria were observed on an inverted microscope (DMIRBE, LEICA, Germany), equipped with an EB-CCD Camera (14 µm pixel, C7190-43, HAMAMATSU, Japan). The camera utilized an S23/0.55 condenser equipped with light rings for darkfield imaging (LEICA, Germany) and a 100 W halogen lamp as a light source. Videomicroscopy for tracking was performed using a 20X dry objective (Leica, Germany; NA = 0.4) with an intermediate 1.5X lens. This objective depth of field is about ± 2.4 µm. Images stacks were acquired every 30 s. Each stack was recorded for 10 s at a frequency of 30 Hz for approximately 2000s (Fig. 2).

### Analyzing the bacterial dynamics by darkfield microscopy

A stack of images (294.4 µm × 220.8 µm) was processed with the multi-particles tracking feature of the HCImage software (HAMAMATSU, Japan) to extract the number of motile bacteria and compile the bacteria trajectories. The trajectories were determined as the coordinates of the centroid of the cell body with a tenth of pixel precision and smoothed by a 3-points central moving average to reduce the position noise. The speed of each bacterium was characterized by the median value of all speed values along its trajectory. It was compiled from the coordinates over 3 points along the trajectory. The trajectories shorter than 4 µm and 1 s or with a mean speed slower than 7 µm s^−1^ were rejected in order to eliminate non-swimming and stuck bacteria. The dynamics of a swimming population was determined with R software and was characterized by the median speed, V, and the quantiles Q25, and Q75, for each stack. The bias of the orientation of the motion of a bacterium was defined as the displacement along the x or y-direction versus the full length of the trajectory recorded during the whole stack (over 10 s), *l*. It was compiled for each trajectory from the coordinates over 5 points vector. The norm ($l$), abscissa ($dx$) and ordinate ($dy$) of this vector were determined to further compile the bias in x ($\sum dx/l$) and in y ($\sum dy/l$) per trajectory, equivalent here to the fraction of velocity drift on velocity ($\sum (dy/t)/(l/t)\sim {v}_{c\_y}/v$). The bias (${\delta }_{x}$) and (${\delta }_{y}$) of the motion of a swimming population was characterized by the median value of the bias of all trajectories and further smoothed over 3 stacks and the velocity drift along the y-axis compiled as ${V}_{c\_y}={\delta }_{y}\times V$.

## Results and discussion

### Videomicroscopy and analysis of individual parameters

The behavior of the bacteria was monitored in a 100 µm-thick channel, filled with a diluted solution of an *E. coli* strain with wild-type swimming behavior (OD_600 nm_ = 0.1) (Fig. 1A, Materials and methods). They were suspended in a tryptone-rich medium for which circular surface swimming was well-characterized [29]. Bacterial density is invariant over time when the well is filled with ultra-pure water only, so neither the introduction of water into the well, nor gas exchange with the air, nor metabolic activity in the channel are potential sources of chemo-effective gradients (Fig. 2A). The experiment was triggered by filling one of the extremity wells with 4 µl of a highly concentrated (250 mM) solution of nickel nitrate suspended in ultra-pure water. Observations were carried out at a fixed position, on the bottom surface, for about 40 min, a duration shorter than the bacterial division time (doubling time ~ 60 min at T = 32 °C). The individual dynamics was analyzed every 30 s and followed as statistics of the speed, radius and directional biases analyzed from the trajectories of the swimming bacteria (Fig. 1B, C). These parameters are described in an orthonormal coordinate system where the z-axis is normal to the surface. The y-axis is parallel to the main direction of the gradient propagation in the, channel and positively oriented in the opposite direction to the well. The x-axis is perpendicular to the y-axis and parallel to the surface.

### Wave formation with nickel nitrate

**Fig. 3:**
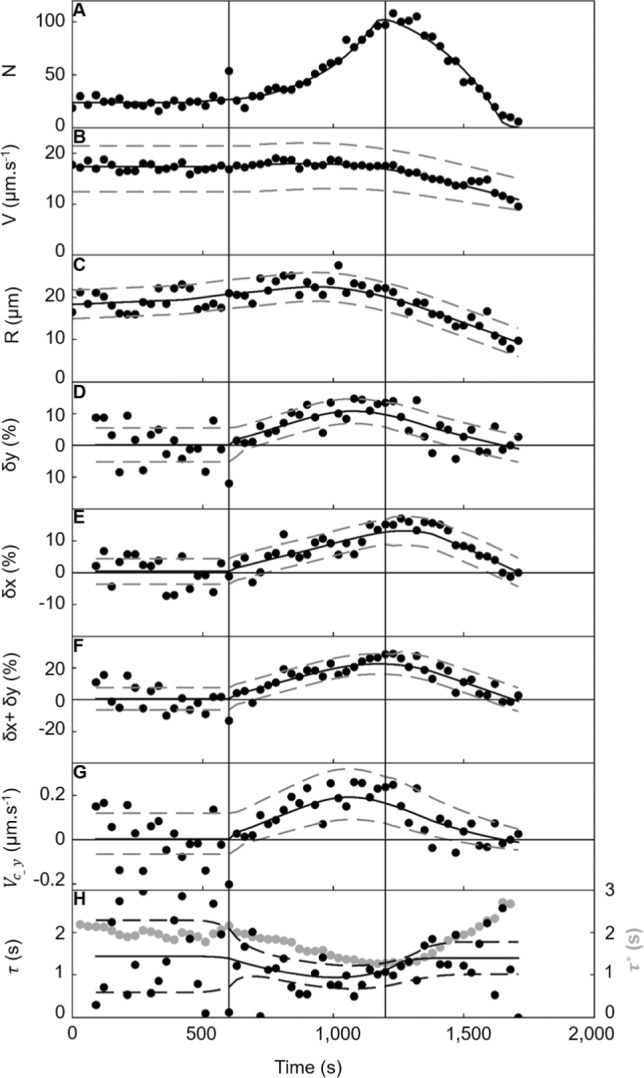
Dynamics of *E. coli* at the surface. **A** The number of bacteria in the observed image, N, swimming at the bottom surface of the channel after repellent addition at t = 0 s versus time. **B** The median speed, V, in an envelope defined by the speed values of the first and third quartiles of the speed distribution. **C** The median radius, R, **D** the median bias per trajectory along the y-direction, ${\delta }_{y}$, **E** the median bias per trajectory along the x direction, ${\delta }_{x}$, **F** the sum ${\delta }_{x}+{\delta }_{y}$, and **G** the velocity drift are in envelopes defined by the SD of the values. **H** Persistence time, $\tau$, is the duration required to cover an arc of a circle of radius R at speed V, which produces biases ${\delta }_{x}$ and ${\delta }_{y}$. Mean duration of trajectory, $\tau *$ (Figs. S2) is reported in grey

The time evolution of the bacteria number showed that bacteria form a wave that propagated away from the repellent source (Figs. S1 and 2). In a first phase (t < 630 s in Fig. 3A), the bacteria number drew a low and constant baseline (N = 25.4 ± 4.4, mean ± SD), identical to the one observed before the repellent addition and without any repellent (Fig. 2A). In a second phase, it starts to increase from baseline forming a ramp that is followed by the emergence of the upward front of a peak. This peak is about 600 s long and four times more intense than in phase #1 baseline. Final bacteria number values passed under the baseline and decreased to close to zero when no bacteria were moving anymore, and few were immobilized in the field of view.

### Individual bacterial dynamics

**Fig. 4:**
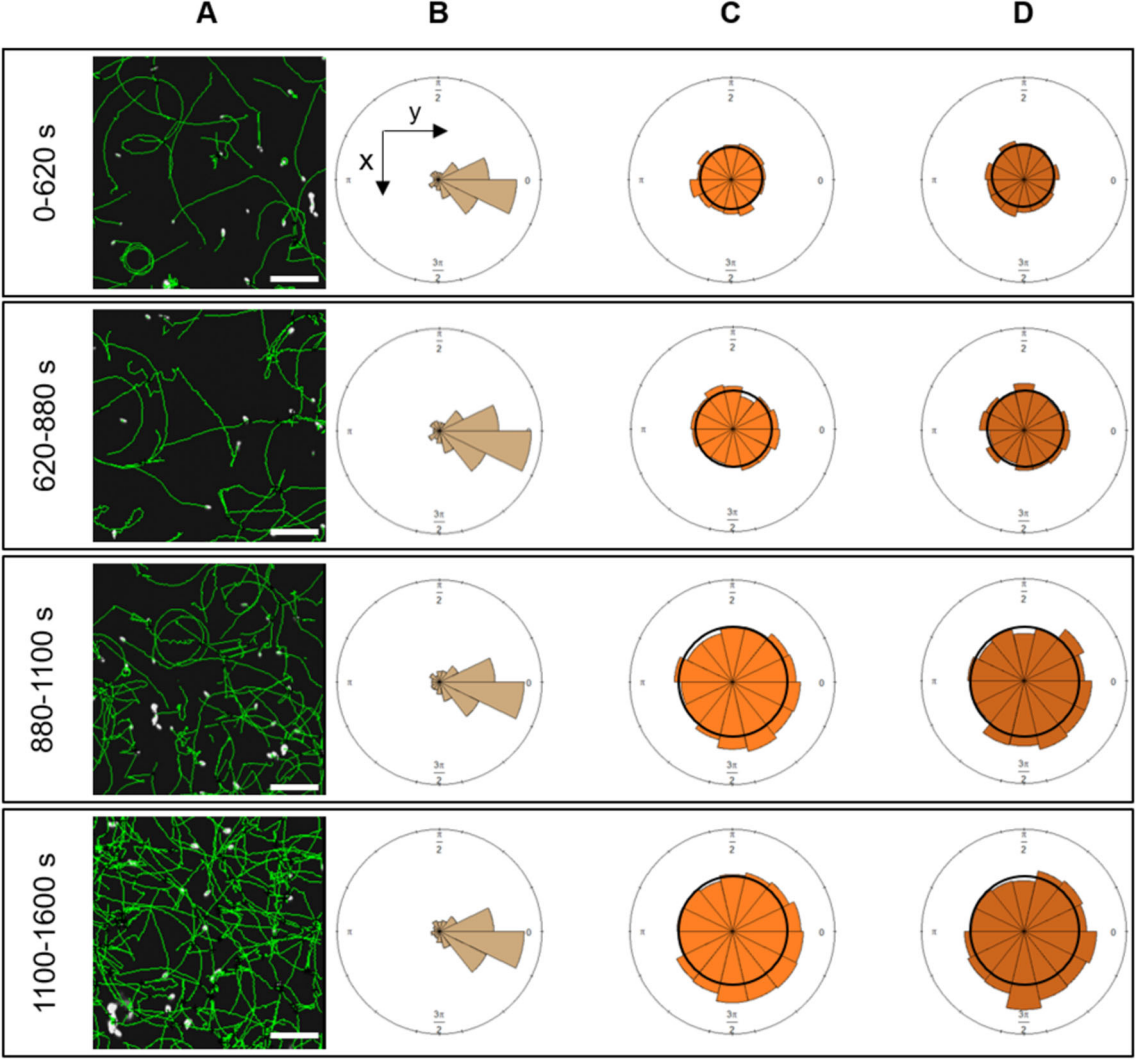
Angular dynamics of *E. coli* at the surface. Study in periods (0–620 s), (620–880 s), (880–1100 s) and (1100-1600 s). **A** The trajectories (green) of the swimming *E. coli* near the surface followed by 2D tracking microscopy during 10 s, are superimposed on the bacteria (white) of the first frame of the acquisition. Scale bar = 50 µm. **B** Rose diagrams of the relative angle reorientation from the first frame (brown) to frame 15 (after 0.5 s) of the trajectories of the swimming bacteria (brown). **C** Rose diagrams of the absolute orientation angle at the first frame (t_0_) of all ith-trajectory, $\bigcirc\!\!\!\!\!\!\text{----}$i(t_0_) (light orange) of the swimming bacteria. **D** Rose diagrams of the absolute orientation angle at the frame 15 after 0.5 s (t_0.5 s_) of all trajectories, $\bigcirc\!\!\!\!\!\!\!\!-\!\!\!-$i(t_0+0.5 s_) of the swimming bacteria (dark orange). **C** and **D** show the overall absolute rotation of the bacteria population. Rose diagrams areas are proportional to the square root number of data; bin size = $\frac{\pi }{7}$

In phase #1, the speed (17 ± 9 µm s^−1^, mean ± SD) and radius distributions of the population (19 µm, mean within a [Q25; Q75] range of [8 µm; 48 µm] (Fig. 1B–E), remain constant over time (Fig. 3B, C). They are characteristics of bacteria surface swimming in the absence of repellent (V = 17.4 ± 0.3 µm s^−1^, R = 22 ± 2.2 µm, mean ± SD). The average values of the bias along the x-direction and the y-direction are null [35] (Fig. 3D, E), this was also observed in the angular distribution (Fig. 4C, D, top panel). In phase #2, speed, radius and bias values started to increase (Fig. 3B–E). The speed value reaches its maximal value in the middle of the upward front of the peak, after which it dropped to 10 µm s^−1^. The values of the bias along the y-direction, the x-direction and their sum increased till the peak (maximum δ_y_ value of ~ 15% in Fig. 3D, E), after which they decrease. The sum of the drifts $\left( {\delta_{x} + \delta_{y} } \right)$ reaches a maximal value on the wave’s peak (32 ± 18% in Fig. 3F), close to the maximum reported values [34].

### Surface conversion of the bacterial population drift δ_y_ into δ_x_ drift.

The wave population shows a notably increased drift in the propagation direction ($\delta_{y}$ > 0, Fig. 3D, F and ${V}_{c\_y}>0$ Fig. 3G), with a mean value at the wave peak of ~ 11 ± 2% (n = 5). As a swimming bacterium comes within 2 µm of a solid surface, hydrodynamics causes its trajectory to curve clockwise [14], converting some of the drift along the y-axis into perpendicular drift along the x-axis. The positive perpendicular drift of the wave population (${\delta }_{x}\hspace{0.17em}$> 0 and ~ 15 ± 0.8% for n = 5, Fig. 3E) indicates that bacteria trajectory bending does not fully randomize the bulk drift in the propagation direction (Fig. 4). This stands in contrast from a previous study involving a gradual, shallow linear chemical gradient of attractants [35].

High drift values occur across various parameters [24]. The mean arc length, calculated as the mean duration of a trajectory, τ* (Figs. S2 and 3H) times the median speed, corresponds to less than a quarter of the perimeter of a model circular trajectory of radius the median radius in the wave peak (Figs. S2). Therefore, the ratio between the model arc length swum at mean speed V during a persistence time, τ, and the perimeter of the circular trajectory with mean radius R which convert the bulk drift into the observed one at the surface can be related to ${\delta }_{x}$/${\delta }_{y}$ as follows:

1 $$ \frac{{\left( {V \times \tau } \right)}}{2\pi R} = \frac{{\left( {\uppi /2 - \tan^{ - 1} \left( {\delta_{x} { }/{ }\delta_{y} } \right)} \right)}}{{2\uppi }} $$

In phase #1, this persistence time shows wide scattering (τ = 1.4 ± 0.9 s, mean ± SD), but in phase #2, it diminishes, reaching a minimum value at the wave peak of approximately 1.1 ± 0.1 s (Fig. 3H). Tumbling events directed towards the bulk terminate trajectories at the surface, while those directed towards the surface are difficult to detect due to minimal changes in orientation. Persistence time in the wave is notably shorter than the tumbles period [15, 16] and residence time values (~ 5 s in [39], ~ 3 s in [35]) reported on the surface without a chemoeffector. The alignment of the trend of the decrease of the duration of the trajectory in the wave (τ* = 2 ± 0.2 s, mean ± SD in phase #1, and 1.6 ± 0.2 s in phase #2 respectively) with that of the persistence time supports that lateral drift of the population is primarily driven by increased tumbling frequency induced by chemorepellent presence.

## Conclusion

On a solid surface, population lateral drift results from a combination of the near-surface ${\delta }_{y}$ drift with a heightened tumbling. Therefore, bacterial migration towards a higher concentration of attractants, likely with long persistence, may not be equivalent to migrating away from a higher concentration of repellents, likely associated with a higher tumbling frequency due to chemorepellent presence. This study sheds light on bacterial survival strategy in response to heightened environmental toxicity. This strategy involves two distinct processes, with contrasting effects on bacterial density. The repellent chemotaxis prompts the escape of the entire population of motile bacteria into a wave, minimizing individual toxin exposure. Meanwhile, at the surface, hydrodynamics and steric interactions of the swimming bacteria drift them laterally, possibly enabling broader exploration and facilitating surface colonization at optimal locations.

## Supplementary Information

Below is the link to the electronic supplementary material.

Supplementary file1 (DOCX 236 KB)

## Data Availability

The authors will provide the data on reasonable request.
