# Supplementary material for: Surface conversion of the dynamics of bacteria escaping chemorepellents
Source: Eur Phys J E Soft Matter. 2024 Sep 15;47(9):56. doi: 10.1140/epje/s10189-024-00450-7 (PMC11402855; doi:10.1140/epje/s10189-024-00450-7)
Supplement: Supplementary file 1 — Supplementary file1 (DOCX 236 KB) [file 10189_2024_450_MOESM1_ESM.docx]

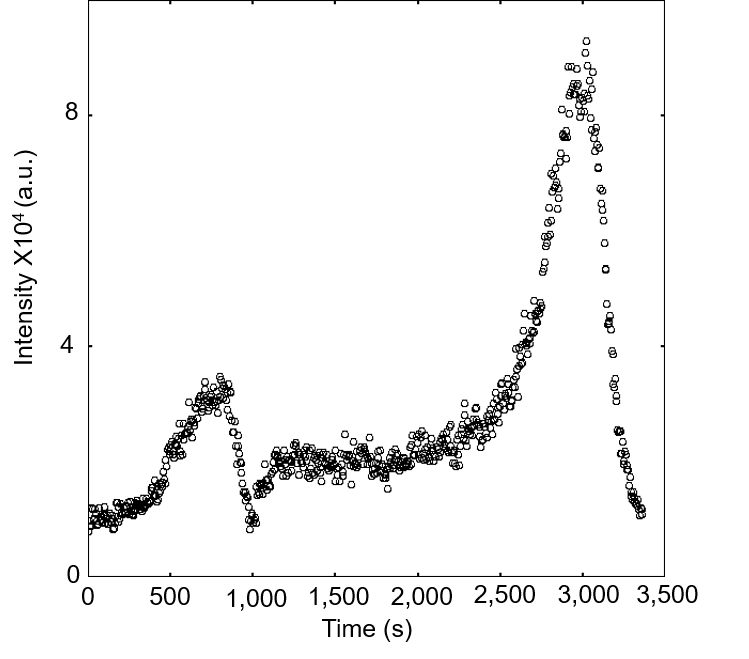


Fig. S1. **The propagation of an *E. coli* wave at the surface of the channel.** Integrated intensities of an image versus time of the bacteria at the surface of the microfluidic channel after repellent addition at t = 0 s recorded at two different positions (y = 3.8mm, wave passage at 800s, and y = 7.8 mm wave passage at 2980s). Concentrations of the Ni(NO_3_)_2_ solution was 250 mM.


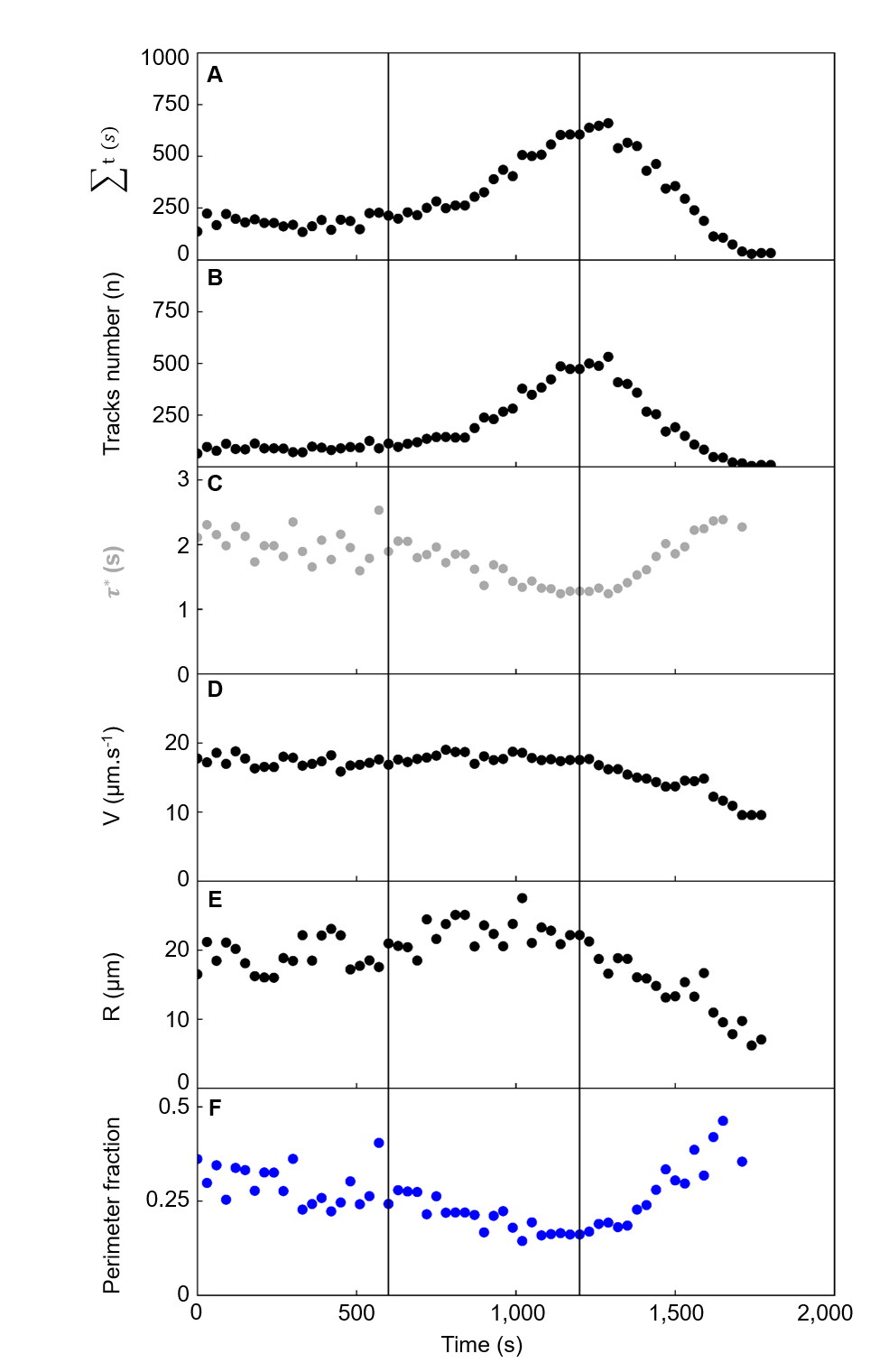


Fig. S2. **Dynamics of *E. coli* at the surface.** (A) The total duration of swimming in a stack of 10s, ∑t. (B) The number of trajectories in a stack of 10s, n, swimming at the bottom surface of the channel after repellent addition at t = 0 s versus time. (C) the mean duration of a trajectory, τ*, calculated as ∑t/n. (D) The median speed, V, as reported in Fig. 3. (E) The median radius, R as reported in Fig. 3. (F) The ratio between the model arc length for a bacterium that swims at mean speed V during a mean duration, τ*, and the perimeter of the circular trajectory with mean radius R, calculated as $\frac{\left( V\times\tau* \right)}{2\pi R}$.
